# Welcome to the 2019 Edition of Belgian Society of Radiology Annual Meeting

**DOI:** 10.5334/jbsr.1914

**Published:** 2019-11-16

**Authors:** Geert Villeirs

**Affiliations:** 1 UZ Gent, BE

Dear Colleagues,

This year’s edition will focus on **Pelvic Imaging** and **Leadership, Management and Quality**, featuring parallel sessions organized by the BSR Scientific Council and the Young Radiologist Section. After lunchtime, a short **Plenary Session** will provide some background about the current status and future directions in Belgian radiology, directly followed by an interesting session on **Radiation Protection** and **Contrast Media Safety**. National and international experts in the field will share their expertise and opinion on these topics in both cutting-edge lectures and refresher courses.

I gladly take the opportunity to particularly thank, once again, Professor Raymond Oyen, President of the Scientific Council, and Drs. Nicolas Devos and Bernard Crutzen, Presidents of the Young Radiologist Section, and all others involved in the organization of this Annual Meeting.

On a personal note, today’s meeting is a special one, both because it will focus on Pelvic Imaging, my personal area of interest, and because it is the last one under my presidency. I have had the honour and privilege to serve our society for over six years and it is now time to pass the torch. Elections for a new board will be organized early 2020 and a new president will be appointed after the General Assembly in February 2020. I gratefully thank the Board Members of the BSR for the wonderful teamwork during all these years and each and every one of you for your confidence and support.

I wish you an interesting and pleasant BSR Annual Meeting 2019!

Warm regards,

Prof. Dr. Geert Villeirs

BSR President

